# MQTree: Secure OTA Protocol Using MQTT and MerkleTree

**DOI:** 10.3390/s24051447

**Published:** 2024-02-23

**Authors:** Yunje Shin, Sanghoon Jeon

**Affiliations:** 1 Department of Software, Kookmin University, Seoul 02707, Republic of Korea; ioerts@kookmin.ac.kr; 2 Department of Automobile and IT Convergence, Kookmin University, Seoul 02707, Republic of Korea

**Keywords:** OTA, Software-Defined Vehicle, V2X, MQTT, Merkle tree

## Abstract

The escalating advancement in Software-Defined Vehicles (SDVs) necessitates a formidable strategy for firmware updates, where traditional methods often fall short of guaranteeing absolute integrity. Although decentralization has been explored in studies for firmware integrity verification using blockchain technology, it lacks comprehensive validation in the context of automotive over-the-air (OTA) updates. By recognizing the limitations of current practices and the partial validation of decentralized approaches, such as blockchain, in the automotive sector, our study introduces a novel mechanism for firmware over-the-air (FOTA) updates. This mechanism is grounded in the widely adopted message queuing telemetry transport (MQTT) protocol, integral to the Internet of Things (IoT) domain, and leverages Merkle tree-based blockchain verification to fortify the fidelity and efficiency of firmware updates. Our proposed solution not only prioritizes the stability crucial to automotive OTA updates but also ensures that performance is not compromised. This dual focus on reliability and efficiency represents a significant stride forward in the development of secure, scalable SDV firmware update protocols.

## 1. Introduction

As vehicles transition to Software-Defined Vehicles (SDVs), their architecture undergoes a shift to a centralized model, accompanied by growing efforts to implement over-the-air (OTA) firmware updates through vehicle-to-everything (V2X) technology. Firmware OTA (FOTA) for the electronic control unit (ECU) of a vehicle’s drivetrain is crucial for the future of connected cars. This technology enables the resolution of recall-level issues without requiring a visit to a service center. OTA, as a broader concept, facilitates seamless software or firmware updates for devices, ensuring that users can easily maintain the latest software versions. FOTA specifically pertains to the OTA process for updating the firmware of hardware modules, such as a vehicle’s ECU. OTA is intricately related to security concerns, given the potential exposure to various threats during the OTA update process. This is particularly evident in the risk of accessing internal networks through a vehicle’s infotainment system [1]. In order to counter these security vulnerabilities, there is a growing adoption of security-enhanced OTA update methods, such as secure OTA.

### 1.1. Problem Statement

Recent research [2,3] has proposed a secure FOTA update approach for ECUs within the in-vehicle drivetrain. This method uses vendor signatures, metadata, and timestamps for each ECU firmware. However, ensuring the complete integrity of the firmware poses challenges because of the ease of forgery. The integration of blockchain has significantly contributed to enhancing firmware integrity and reliability. Therefore, there is a notable focus on research on secure FOTA using blockchain to secure firmware integrity [4]. Blockchain’s decentralized integrity verification for firmware updates relies on consensus algorithms. However, because of the safety-critical nature of automotive OTAs, the application of FOTA using blockchain has not yet been sufficiently validated for real-world vehicles. This is because it does not eliminate the potential impact of security issues or incorrect uploads in a distributed network architecture on the entire vehicle update network.

Moreover, implementing OTA using the HTTP or HTTPS (or TLS) protocol based on the centralized network architecture, which is the most widely used in web communication, offers advantages in terms of ease of use and high compatibility. However, it is characterized by high overheads and low efficiency because of its disconnected nature. In particular, HTTPS requires a separate transport layer security (TLS) handshake process for each request and response. While enhancing security, this process introduces additional processing and data transfer time by involving multiple steps, including exchanging encryption keys, authenticating the server, and establishing a secure session for each HTTPS connection. This process can result in considerable overhead, especially in environments with frequent short requests and responses. Therefore, numerous studies have employed message queuing telemetry transport (MQTT), a lightweight protocol that is widely used in the Internet of Things (IoT), to reflect the characteristics of automotive OTAs, where both safety and OTA update speed are crucial considerations [5]. MQTT is a protocol designed for continuous and periodic message exchange, such as in an IoT environment. It establishes a secure connection with a single TLS handshake and then sustains data exchange without additional handshakes once the connection is established. This characteristic results in relatively low overhead when exchanging messages.

For HTTPS, addressing some of this overhead is possible by using session reuse. This involves saving the results of a TLS handshake and reusing them when reconnecting to the same server, eliminating the need for a new handshake. While this can accelerate connection times and reduce overhead, it can still result in a relatively high overhead compared with MQTT. This is attributed to the inherent nature of HTTPS, which requires a separate connection for each request and response. In conclusion, HTTPS and MQTT are designed for different environments and purposes, which results in varying amounts and types of overhead. HTTPS excels in web-based communication and manages overhead through session reuse, whereas MQTT efficiently handles communication in environments such as IoT through persistent connections [6]. This study adopts MQTT with TLS to combine the security of TLS’s centralized network architecture with MQTT’s performance-enhancing traits, which are prevalent in the IoT. The study introduces and implements *MQTree*, a secure OTA technique that uses Merkle Tree, a blockchain verification technology, over MQTT (with TLS) to improve firmware integrity and reliability.

### 1.2. Main Contributions

The contributions of this research are as follows:

- The proposal of a novel secure OTA technique, *MQTree*, which uses MQTT (with TLS). This technique combines the advantages of HTTPS and MQTT protocols within a centralized network architecture to improve safety and performance in automotive OTA updates simultaneously. It incorporates Merkle tree, a blockchain verification technology, to improve firmware integrity and reliability.
- The integration of MQTT (with TLS) and Merkle tree, revealing a substantial reduction in processing time and payload-related overhead while maintaining security compared with the existing HTTPS-only OTA method. In our experiments, *MQTree* demonstrated a performance improvement of approximately four times the processing time compared to HTTPS alone.
- The open-sourcing of *MQTree* [7], the secure OTA technique proposed in this study, to enable related researchers to replicate its results and incorporate them into their own research.

### 1.3. Scope

This study concentrates on the communication protocol aspect within the three key areas of secure OTA: security at the end device, the approach to firmware updates within the end device, and the communication protocol. In essence, this study emphasizes securing the network and operates on the premise that end-device security is achieved through a hardware security module (HSM). Furthermore, it is assumed that server-side security is impenetrable, underpinned by robust security measures, such as multi-factor authentication (MFA), the principle of least privilege (PoLP), and security information and event management (SIEM) systems. Therefore, this study focuses on implementing TLS into the MQTT communication protocol to enhance the security of data transmission over the network. TLS usage ensures the confidentiality and integrity of data through encryption during transmission. Essentially, the study combines the MQTT and TLS protocols to capitalize on the strengths of both. This combination is the first step in enhancing network security and is further fortified by TLS encryption during data transmission, which plays a crucial role in ensuring the confidentiality and integrity of data. However, security systems are perpetually susceptible to potential threats, and the possibility of TLS being compromised remains a reality. In this study, we incorporate Merkle trees as an additional layer of security. A Merkle tree is a structure that can effectively detect instances where data have been compromised or altered during transmission over a network. In the event of a TLS security breach, the Merkle tree is used to verify data integrity and promptly detect any corrupted or altered data. Therefore, this study proposes a robust and comprehensive security scheme that can cope with the various security threats that may occur during data transmission over a network.

### 1.4. Paper Organization

The remainder of the paper is organized as follows: Section 2 introduces the background, detailing MQTT and Merkle Tree technologies and their relevance to OTA updates. Related work is reviewed in Section 3. Section 4 delves into the proposed method, breaking down the firmware uploading, publishing, and in-vehicle operations. The evaluation of the method, including security verification and performance validation, is presented in Section 5. Section 6 engages in a discussion about the study’s implications, limitations, and potential future work. Finally, Section 7 concludes the paper, summarizing its contributions and findings.

## 2. Background

In this section, we describe the key technologies used by *MQTree*, namely MQTT and Merkle Tree, and provide a rationale for their selection. Subsequently, we explore the major threats associated with OTA updates. Finally, we outline the scope of this study.

### 2.1. MQTT

**Figure 1:**
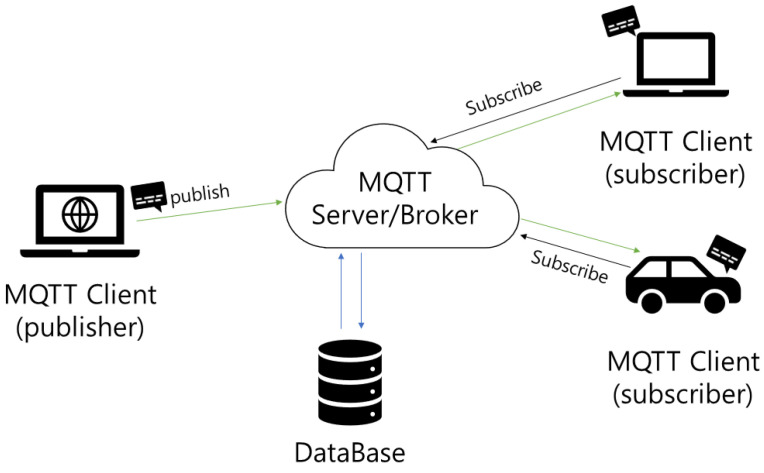
The MQTT Protocol.

Figure 1 illustrates the MQTT protocol, which provides fast performance due to its lightweight structure and efficient message transfer mechanism. The performance and reliability of this protocol are predominantly attributed to the following features and mechanisms.

- Lightweight protocol structure: MQTT employs a very simple header with a fixed length of 2 bytes. This minimizes the protocol’s overhead and enables efficient data transfer in low-bandwidth environments. In addition, MQTT operates on top of the TCP/IP protocol, thereby leveraging the reliability of network connections.
- Publish and subscribe model: MQTT adopts a publish and subscribe model, which eliminates strong ties between message publishers and subscribers. A publisher publishes a message to a specific “topic”, and only subscribers who subscribe to that topic receive the message. This model increases the efficiency of message delivery and provides scalability on large networks.
- Quality of service (QoS) support with three levels. First, QoS-0 (at most once) ensures messages are sent at most once, enabling swift delivery in situations where delivery confirmation is not required. Next, QoS-1 (at least once) guarantees message delivery at least once, enhancing reliability for crucial messages, albeit with a potential for duplicates. Finally, QoS-2 (exactly once) ensures that messages are delivered exactly once, offering the highest reliability but involving additional processing and overheads. For example, while QoS-1 involves sending a message and receiving an acknowledgment via a PUBACK packet, QoS-2 entails a more complex four-step handshake to ensure that the message is delivered exactly once.

Given the characteristics of MQTT, supporting secure OTA updates tailored to specific requirements at the protocol level becomes feasible. This approach provides a potent means to achieve both secure and efficient firmware updates in IoT environments.

### 2.2. Merkle Tree

**Figure 2:**
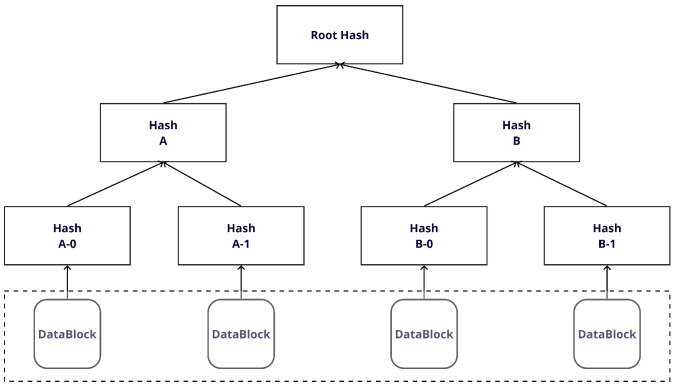
The Merkle Tree.

A Merkle tree, which is a crucial data structure in blockchain technology, assumes the form of a binary tree. Each node in this tree stores the result of its children’s hashed values. Through this mechanism, the Merkle tree ensures the integrity of each node and maintains the consistency of the entire data structure. When employing a Merkle tree for firmware updates, as shown in Figure 2, the process begins by reading all firmware files into small chunks, and hashes are computed for each chunk. These hashes form the leaf nodes of the Merkle tree. The parent nodes then rehash the hashes of their respective children and store the results. This iterative process continues until it reaches the top node of the tree, which is known as the root. A notable strength of Merkle trees lies in their ability to propagate changes. If a single chunk of data changes, it affects the entire tree. A change in the data or hash value of one chunk triggers a change in the hash value of all parent nodes containing that chunk. Consequently, the root hash stored at the root node undergoes a change, simplifying the detection of any alterations or manipulations to the data. These characteristics enable Merkle trees to significantly contribute to ensuring the integrity and reliability of the firmware during the firmware update process.

### 2.3. Major Attacks for OTA Updates

To identify the main attacks that can occur during the OTA update process, we analyzed the security threats and attack types associated with MQTT and IoT. The examination drew insights from studies by Palmieri et al. [8], Sahlmann et al. [9], and Hintaw et al. [10]. These studies identified various attack types within OTA protocols, categorizing them as DoS/DDoS, man-in-the-middle (MITM), spoofing, and duplicate update. The selection of these attack types stems from their frequent occurrence in MQTT and IoT environments, as well as their substantial impact on the stability and reliability of IoT systems from a network perspective. Primarily, denial of service (DoS) and distributed DoS (DDoS) attacks deplete system resources and disrupt services. In an MITM attack, an attacker intercepts communication by tampering with or eavesdropping on messages. Spoofing attacks involve an attacker impersonating another user or system to obtain or transmit data illegally. Lastly, duplicate update attacks disrupt normal system operation by allowing an attacker to resend an update message that has already been transmitted. Although the MQTT protocol is optimized for lightweight messaging, it lacks a default feature for the automatic detection and handling of duplicate message sending. This introduces the risk of mistaking duplicate update messages for original messages. For example, if an attacker captures and resends a firmware update message, the system may mistakenly perceive an already applied update as new and re-apply it. This scenario can result in unnecessary resource consumption, system instability, and potential malfunctions. Table 1 summarizes the main attacks that can occur during OTA updates.

## 3. Related Work

Research on automotive OTA update systems can be broadly categorized into two perspectives: protocol and end-device. As shown in Table 2, this study specifically focuses on the protocol perspective and divides the research into two sections: research on communication reliability and data transfer efficiency and research on security enhancement. This categorization aims to systematically examine two crucial aspects of OTA update systems: efficiency and security. First, the research on the reliability of communications and the efficiency of data transfer focuses on enhancing the efficiency and reliability of data transfer in OTA systems. It explores the optimization of protocols and technologies essential for swift and reliable data delivery within a network environment. This includes the use of lightweight communication protocols, such as MQTT, and methods to improve their security. Meanwhile, the research on security enhancement focuses on ways to enhance the integrity and safety of data. It explores ways to enhance data protection during firmware updates and overcome vulnerabilities in centralized systems. This approach encompasses the use of distributed database solutions such as blockchain technology. This study integrates these two areas, with a particular focus on protocol-based approaches. This combined approach contributes to simultaneously improving the reliability and efficiency of automotive OTA update systems and plays a key role in developing effective solutions that respond to different needs.

### 3.1. Research on the Reliability and Efficiency of Communications

In the realm of IoT environments, MQTT stands out as one of the most popular protocols. According to a study conducted by Gemirter et al. [11], MQTT exhibits performance comparable to that of the advanced message queuing protocol (AMQP) but with significantly shorter message transfer times and lower CPU use than HTTP. This underscores MQTT’s advantages in terms of efficient message delivery and resource use when transmitting continuous update packets. Luzuriaga et al. [12] further demonstrated the superiority of MQTT over AMQP in maintaining the order of message delivery in unstable environments, such as vehicular networks. This attribute is especially useful in scenarios where maintaining sequential packet reception is crucial, such as OTA updates. A study conducted by Thantharate et al. [13] compared CoAP and MQTT-based models and revealed that MQTT exhibits shorter message delivery times than CoAP, even under limited RF conditions. Particularly, at QoS levels 1 and 2, MQTT demonstrated faster delivery rates than CoAP, showcasing its high transmission efficiency across various network conditions. Similarly, Al-Fuqaha et al. [14] evaluated the delay performance of CoAP and MQTT, revealing that MQTT exhibited a lower delay than CoAP under low packet loss conditions. These results underscore the significance of MQTT’s stability and reliability in facilitating swift data transfer and reliability in IoT environments.

Sahlmann et al. [9] introduced an OTA firmware update method using MQTT. Their method slices the firmware file for efficient transmission. In addition, they proposed a method to maintain the integrity of the firmware file and prevent data loss by providing the location of the uploaded firmware file as a URL address in JSON format. In a study by Chandra et al. [15], URLs were securely downloaded over the HTTPS protocol, thereby enhancing data security in IoT environments. In another study, Stoev et al. [16] employed a 256 bit symmetric encryption algorithm to enhance the security of insecure MQTT communication. The study introduced the AES256 ECB mode to enhance MQTT protocol security, but it exhibited vulnerability to replay attacks. The study highlighted various limitations, including the generalization problem of the security mechanism, which potentially causes additional security vulnerabilities. To mitigate these issues, we proposed the use of MQTT with TLS. Although this may slightly affect performance, it is deemed an effective means to enhance the scalability of the existing architecture. Specifically, security is enhanced by implementing a minimal authentication procedure when subscribing to a specific topic. For periodic update packets, MQTT with TLS is employed to ensure reliable and secure data transmission. This integration positions MQTT as a versatile IoT specification that seamlessly combines HTTP and TLS in a TCP/IP-based environment to provide more robust solutions for data transmission and security.

### 3.2. Research on Security Enhancements

Research on blockchain-enabled OTA updates explores decentralized approaches that diverge from the traditional centralized model [17]. Explorations include direct firmware downloads for IoT devices from a distributed database system rather than from the central server [18]. Hash tables and public blockchains are leveraged to ensure firmware integrity and authenticity for OEMs. These efforts aim to enhance MQTT security using blockchain as a novel solution to the security challenges of the IoT [19]. Researchers have attempted to leverage the Ethereum blockchain to address basic security vulnerabilities in the MQTT protocol [20,21]. Their proposition involves an architecture based on smart contracts that facilitates transaction control and storage between clients and brokers to enhance security while maintaining the existing architecture as closely as possible. More recently, a blockchain-based approach has been proposed to fortify MQTT communication security using a sharding technique to partition data into smaller segments, which are distributed across multiple database servers [22,23]. This approach is suitable for resource-constrained IoT environments and uses the smart contract mechanism of the Ethereum blockchain to ensure trust, accountability, and user privacy. The inherent tamper-proof nature of blockchain data suggests several means to reinforce the security of data stored on the broker. Although blockchain proves effective for maintaining the integrity and reliability of frequently updated firmware, a drawback lies in the need for a decentralized structure from the vendor, which requires substantial time and effort to ensure the safety of distributed nodes. The core of existing research is to leverage blockchain to enhance security while maintaining the existing network architecture as much as possible, but this practically means moving away from a centralized approach. The introduction of blockchain involves a fundamental change in the existing architecture, which goes beyond simple security enhancement and leads to a change in the system structure itself. However, rather than exploring a decentralized approach by breaking away from the manufacturer-centric centralized approach, this paper continues to use the Merkle Tree while maintaining a centralized approach to address these issues. By using the Merkle Tree, the reliability and integrity of the firmware can be guaranteed while maintaining the efficiency and stability of the centralized structure. This approach has the advantage of reducing the complexity and cost of a blockchain-based decentralized approach while still achieving the high level of security required during the firmware update process.

The primary focus of existing research is to use blockchain for heightened security while maintaining the existing network architecture, indicating a departure from a centralized approach. Introducing blockchain implies a fundamental change in the existing architecture, which extends beyond simple security enhancement to a restructuring of the system itself. However, rather than exploring a decentralized approach by deviating from the manufacturer-centric centralized approach, this study continues to use the Merkle tree while maintaining a centralized approach to address these issues. The Merkle tree ensures the reliability and integrity of the firmware while maintaining the efficiency and stability of the centralized structure. This approach offers the advantage of minimizing the complexity and cost to the level of a blockchain-based decentralized approach while still achieving the high level of security required during firmware updates.

## 4. Main Method

**Figure 3:**
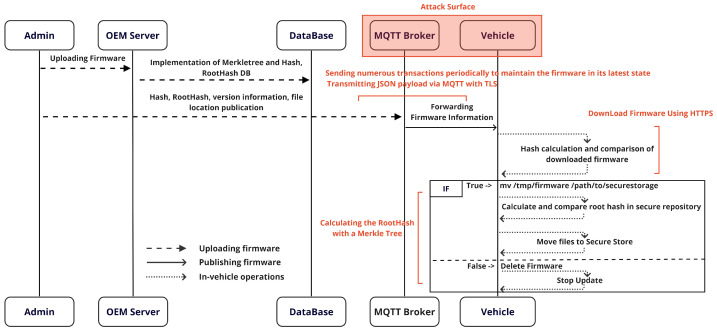
An overview of the MQTree process.

This section outlines the detailed implementation of the *MQTree* OTA firmware update process proposed in this paper. In this process, firmware upload and issuance operations consistently occur sequentially, with SHA-2 being the recommended hash algorithm for security. However, the choice of hash algorithm can be determined based on considerations such as security requirements, compatibility, and computational efficiency. In order to further enhance security, a whitelisting approach is used to manage the firmware upload and download processes. This plays an important role in preventing unauthorized access and increasing the reliability of firmware updates. Figure 3 illustrates the overall sequence of the OTA implementation. It systematically describes the firmware upload process, the firmware issuance through MQTT, and the subsequent behavior in the vehicle (client). This sequence provides a crucial foundation for ensuring the efficiency and safety of the OTA update process. The integrated operation of each phase of the OTA update system is described, emphasizing the significance of the security measures applied to each phase, which play an important role in improving the reliability and efficiency of the entire system.

The subsequent subsections describe the OTA update process of *MQTree* in sequential order: uploading firmware, publishing firmware, and in-vehicle operations.

### 4.1. Uploading Firmware

Ensuring the safe management and security of firmware requires the department responsible for a specific MCU to upload firmware files with authentication and authorization rights. This preventive measure safeguards against unauthorized uploads or tampering. During this process, the uploaded firmware files are stored on a server, where they are used to create a Merkle tree.

**Figure 4:**
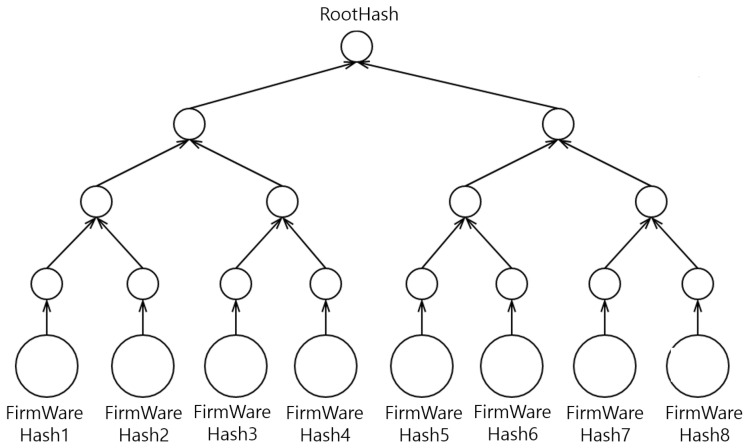
Hash generation mechanism in the Merkle tree.

Figure 4 illustrates the organization of the Merkle tree based on the unique hash value of each firmware file. The structure is pivotal in verifying the integrity of the firmware because even the slightest change in the file’s content alters its hash value. Each leaf node in the Merkle tree represents the hash value of an individual file, whereas each parent node obtains a new hash value generated through the combination of the hash values of its child nodes. The hash value at the top node of the Merkle tree, which is the root node, becomes the root hash representing the entire tree. This root hash can be stored in a database to effectively manage and verify the overall integrity and authenticity of firmware files.

Attacking the root hash of the Merkle tree in the *MQTree* presented in this paper involves overcoming numerous complex and practically insurmountable hurdles. First, the attacker must change the root hash of the Merkle tree on both the server and the client side. This requires deep access to both systems and a high level of hacking proficiency, and typical security systems prohibit such attempts. Second, on the client side, advanced security devices, such as HSMs, must be bypassed. HSMs are securely designed hardware for key management and cryptographic operations and are highly resistant to bypass attempts. Finally, from the network protocol perspective, the attacker must decrypt encrypted network protocols such as TLS, which is known for its strong encryption in securing data transfers. Cracking TLS poses significant difficulty with current technology. However, even if TLS is compromised, the subsequent hurdle involves modifying the existing firmware hash value stored in the HSM on the client side to change the root hash, a task that has proved to be challenging in practical terms.

### 4.2. Publishing Firmware

Ensuring security through a centralized approach involves having an update manager separate from the upload manager. This administrator uses the MQTT protocol to issue the hash value, root hash value, version information, and file location of the new firmware to the topic to which the vehicle is subscribed. Access to the topic is authenticated using keys, which are typically in the form of a username and password. These sensitive credentials are securely stored on a storage medium such as a highly secure HSM. HSMs provide a robust level of security, effectively safeguarding key information from potential leaks or unauthorized access. This ensures a secure connection between the vehicle and the MQTT server, allowing access to the subscribed topics. The transfer and update of firmware information are executed with a high level of security and reliability through these enhanced security mechanisms. This stringent approach ensures that vehicles exclusively receive firmware updates from trusted sources and protects the system against unauthorized access and malicious attacks.

### 4.3. In-Vehicle Operations

In this subsection, the vehicle’s operation is divided into distinct steps: firmware download and hash verification in the vehicle, firmware synchronization and RootHash verification in the vehicle, and firmware update in the MCU. Each step is described as follows.

#### 4.3.1. Firmware Download and Hash Verification in the Vehicle

**Figure 5:**
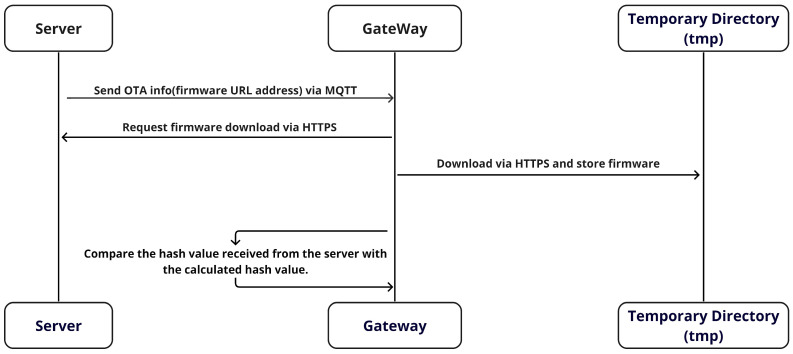
The firmware download process.

Figure 5 shows the process in which the Gateway in the vehicle receives the firmware’s URL address from the server through the MQTT protocol. Subsequently, it proceeds to download the firmware using the HTTPS protocol. While MQTT specializes in transmitting lightweight messages, which makes it suitable for continuous data streams, HTTPS is better suited for sending large amounts of data, such as with firmware, because of its scalability facilitated by various state management codes. In addition, HTTPS offers the ability to break data into blocks and retransmit lost blocks.

HTTPS is an application of TLS to HTTP that provides encryption during data transfer and ensures the confidentiality and integrity of the data. This encryption is crucial for protecting against man-in-the-middle attacks and verifying server authenticity. In addition, it preserves features, such as status codes, which enhance the reliability of network communication and enable scalable web-based communication. After downloading, the firmware file is stored in a temporary directory on the vehicle. The use of a temporary directory safeguards against potential problems during the download and ensures the secure storage of the file. The temporary directory is isolated from the main file system, thereby ensuring that firmware downloads do not affect vital system files or settings. This structure protects critical system components from the potential problems that may arise during the download process. In addition, these temporary directories typically have limited access, which provides an additional layer of security through user-right separation. This permission management ensures that any corruption or unauthorized alteration of the firmware files does not affect the rest of the system. This approach is pivotal in protecting the system from various security threats and creating an environment conducive to promptly detecting and responding to any unusual activity.

The firmware file is processed by dividing it into chunks of 4096 bytes. For each chunk, a hash value is calculated using the SHA-256 hash algorithm, which is important for verifying the integrity of the file. SHA-256 is a strong hash algorithm with minimal risk of hash collisions, making it ideal for this verification. The calculated hash value is then compared with the hash value of the original firmware file transmitted via MQTT. This comparative analysis verifies the accuracy of the firmware, which significantly contributes to enhancing system security. Finally, the gateway monitors the vehicle’s network environment during normal operation, actively assessing the potential for new firmware updates. This involves periodic communication with the server to verify the availability of the latest firmware update. Consequently, the vehicle’s firmware remains consistently up-to-date, continuously contributing to the enhancement of the vehicle’s security and performance. The detailed procedure is described in Algorithm 1.
**Algorithm 1 Firmware download and verification algorithm**1:**procedure***DownloadAndVerifyFirmware*(*URL*)2:    expectedHashValue← ***ReceiveExpectedHashViaMQTT()***3:    firmware← ***DownloadFirmware(URL)***4:    allChunks← *[]*5:    **while** *not EndOfFirmware(firmware)* **do**6:        chunk← ***ReadNextChunk(firmware, 4096)***7:        allChunks.append(chunk)8:    **end while**9:    completeFirmware← ***Concatenate(allChunks)***10:    hashValue← ***CalculateHash(completeFirmware)***11:    **if** *hashValue != expectedHashValue* **then**12:        *throw**VerificationError(“Hash MisMatch”)***13:    **end if**14:    ***StoreInTemporaryDirectory(firmware)***15:**end procedure**

#### 4.3.2. Synchronizing the Vehicle’s Firmware and Verifying the RootHash

**Figure 6:**
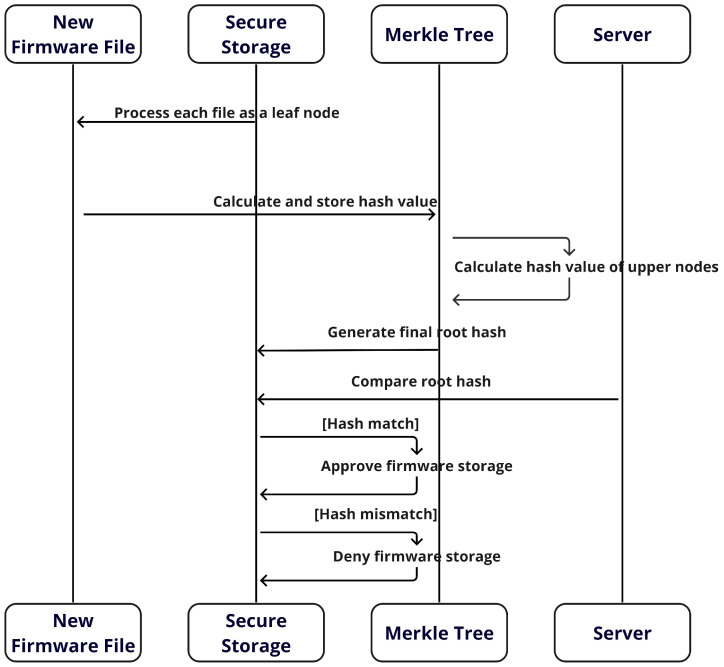
Sequence of procedures for synchronizing and storing firmware in a vehicle.

As shown in Figure 6, following the successful completion of the firmware hash verification process in Section 4.3.1, the vehicle’s secure storage designates each firmware file as a leaf node of the Merkle tree. In simple terms, each firmware file stored in the vehicle corresponds to a leaf node of the Merkle tree, with the hash value of each file calculated and stored in the tree. This methodology facilitates the construction of the Merkle tree, in which the hash values of all leaf nodes are systematically combined to compute the hash values of the parent nodes. This process continues until the root node is reached, and the root hash that is finally generated represents the integrity of all firmware files stored on the vehicle. The root hash computed on the vehicle is compared with the root hash received from the server. If a match is identified, the new firmware is stored in the appropriate secure location. These procedures are pivotal in maintaining the security and integrity of the vehicle’s firmware system. The detailed procedure is described in Algorithm 2. The hash value of the leaf node is calculated by applying a hash function to each firmware file. This can be expressed as follows:

(1) $$L_{i}=H\left(F_{i}\right)$$

$$\begin{array}{cc} L_{i} & \mathrm{Hash}\mathrm{value}\mathrm{of}i-\mathrm{th}\mathrm{leaf}\mathrm{node}\\ H & \mathrm{Hash}\mathrm{function}\\ F_{i} & i-\mathrm{th}\mathrm{firmware} \end{array}$$

**Algorithm 2 Synchronizing and verifying the firmware algorithm**1:**procedure***SynchronizeAndVerifyRootHash*(*firmwareFiles*)2:    expectedRootHash← ***FetchFirmwareFromSecureStorage()***3:    existingFirmwareFiles← ***DownloadFirmware(URL)***4:    combinedFirmwareFiles← ***CombineFirmwareFiles(existingFirmwareFiles, firmwareFiles)***5:    leafNodes← *[]*6:    **for** *each file in combinedFirmwareFiles* **do**7:        hashValue← ***CalculateHash(file)***8:        *leafNodes.append(hashValue)*9:    **end for**10:    merkleTree← ***BuildMerkleTree(leafNodes)***11:    computedRootHash← ***merkleTree.getRootHash()***12:    **if** *computedRootHash != expectedRootHash* **then**13:        *throw**VerificationError(“RootHash MisMatch”)***14:    **end if**15:    ***StoreFirmwareSecurely(firmwareFiles)***16:**end procedure**

A new hash value is generated by concatenating the hash values of neighboring nodes, a procedure that is essential to maintaining integrity. This can be expressed by Equation (2), where $L_{i}$ represents the hash value of the i-th leaf node, $F_{i}$ is the i-th firmware file, and *H* denotes the hash function. Subsequently, the hash values of neighboring leaf nodes are combined to compute the hash value of the parent node.

(2) $$N_{j}=H(L_{i}\;||\;L_{i+1})$$

$$\begin{array}{cc} N_{j} & \mathrm{Hash}\mathrm{value}\mathrm{of}j-\mathrm{th}\mathrm{parent}\mathrm{node} \end{array}$$

where $N_{j}$ denotes the hash value of the jth parent node, and $L_{i}$ and $L_{i+1}$ represent the hash values of the neighboring leaf nodes. Finally, the hash value of the root node is computed by combining the hash values of the parent nodes. This is used to verify the integrity of the Merkle tree.

(3) $$R=H(N_{1}\;||\;N_{2}\;||\;\ldots \;||\;N_{k})$$

$$\begin{array}{cc} R & \mathrm{Hash}\mathrm{value}\mathrm{of}\mathrm{root}\mathrm{node} \end{array}$$

In Equation (3), *R* is the root hash and $N_{1},N_{2},\ldots ,N_{k}$ are the hashes of the top-level nodes.

#### 4.3.3. Firmware Update in MCU

As shown in Algorithm 3, the vehicle’s firmware is updated only after verifying that the vehicle is safely parked. This precautionary measure prevents the firmware update from affecting the vehicle’s operation during real-world driving conditions. In the context of this study, the Jetson Orin, which serves as a central gateway within the vehicle, functions as a central control unit. It uploads the firmware to the Arduino-based MCU using the Arduino command line interface (CLI). The selection of Jetson Orin for uploading firmware to Arduino-based MCUs is primarily for ease of implementation and accessibility. During this process, the gateway first verifies a secure network connection and retrieves the newly verified firmware from secure storage. Subsequently, it transmits the firmware to the MCU, which can be automated using a specially designed script or command set. After completing the firmware upload, the gateway verifies the success of the update and performs additional post-processing if required. This approach ensures the safety and reliability of updating a vehicle’s firmware and contributes to maintaining the vehicle’s performance and reliability.
**Algorithm 3 Firmware update algorithm**1:**procedure** *UpdateFirmwareInMCU*(*firmware*)2:    **if** *not IsVehicleParked()* **then**3:        *return “Vehicle is not in a parked state.”*4:    **end if**5:    userConfirmation← ***GetUserConfirmationForUpdate()***6:    **if** not userConfirmation **then**7:        *return “User has cancelled the firmware update.”*8:    **end if**9:    secureConnection← ***VerifyInternalNetworkConnectionWithECU()***10:    **if** not secureConnection **then**11:        *return “Failed to establish a secure network connection with the internal ECU.”*12:    **end if**13:    ***UploadToFirmware(MCU, firmware)***14:    ***VerifyUpdateSuccess()***15:    ***PerformPostProcessing()***16:**end procedure**

## 5. Evaluation

We designed a series of experiments to evaluate the effectiveness of *MQTree* by investigating the following research questions:

- RQ1. What enhancements in security does the *MQTree* technique offer over HTTPS-only methods due to the incorporation of Merkle tree technology?: The study categorized potential attacks on the OTA protocol into four types: spoofing, MITM, DDoS, and duplicate update. An in-depth analysis was performed to assess the effectiveness of *MQTree* in defending against each of these attacks (refer to Section 5.1).
- RQ2. By what magnitude does the *MQTree* secure OTA technique, which integrates MQTT (with TLS) and Merkle tree technology, improve performance in terms of processing time and payload overheads compared to traditional HTTPS-only methods in automotive OTA updates?: This study also focused on assessing the performance, specifically latency, of the *MQTree*. Comparative measurements were conducted to determine whether *MQTree* enhances or diminishes performance in relation to relevant studies, such as HTTPS and MQTT without TLS (refer to Section 5.2).
- RQ3. What measures have been taken to enable the replication of research results through the open-sourcing of *MQTree*?: The code for *MQTree* proposed in this study has been released on GitHub [7] so that interested researchers can reproduce the results, and an installation manual for configuring the execution environment is provided. This approach supports the open science initiative and encourages collaborative improvements and validations within the research community, particularly in the context of automotive OTA updates.

### 5.1. Security Verification

This section performs security verification for the four security threats defined in Section 2.3: Dos/DDoS, MITM, spoofing, and duplicate update. In addition, we categorize existing works into three groups, as shown in Table 3, to explore effective methodologies that fulfill security requirements by verifying the security of these existing studies. Finally, a comparative analysis was performed to assess and analyze these methodologies.

Table 4 shows a comparative analysis of *MQTree* and related studies in terms of security threats. First, Chandra et al. [15] proposed an OTA firmware update system using the Rest API. This system involves periodic communication between gateway nodes and a server to check for the latest version of the firmware, which is stored in internal storage when necessary. Despite its ability to ensure user identity through TLS and prevent spoofing or man-in-the-middle attacks through encryption, TLS introduces vulnerabilities related to redundant updates, which leads to packet retransmission. Stoev et al. [16] implemented secure communication between ESP8266 Wi-Fi modules using 256 bit AES encryption through the MQTT protocol. They introduced asymmetry in data transmission using a pseudo-random number generator. Although AES ensures data confidentiality, it addresses the issues associated with network traffic or prevents overload situations. The encryption provided by AES encryption makes it difficult for attackers to understand or manipulate the contents, even if the data is intercepted. However, this implementation lacks the ability to verify or authenticate the identity of a user or device. While it allows for simple and effective secure communication, it remains vulnerable to replay and duplicate update attacks, even with a minimized key validity period. In addition, although the MQTT protocol is designed for lightweight messaging, it lacks native features for the automatic detection and handling of duplicate message transmissions. Consequently, there is a potential risk of mistaking duplicate update messages for original messages and processing them as such. For example, if an attacker captures and resends a firmware update message, the system may mistakenly perceive a previously applied update as new and re-apply it. This scenario can lead to unnecessary resource consumption, increased system instability, and potential malfunctions. Therefore, Sahlmann et al. [9] used the MYNO update protocol (MUP) to transmit plaintext messages without relying on TLS and used an edge architecture to mitigate certain risks. However, it cannot entirely prevent MITM, spoofing, redundant update, and DoS/DDoS attacks in the event of a security breach at the edge nodes. Da Silva et al. [24] applied TLS to MQTT to secure the OTA system, but similar to Chandra et al. [15], it has limitations against duplicate update attacks.

In *MQTree*, the use of TLS is crucial for effectively defending against spoofing and MITM attacks. Identity verification through encryption and certificates plays an important role in preventing these types of attacks. In addition, *MQTree* incorporates a Merkle tree-based integrity verification mechanism, which offers an effective defense against duplicate update attacks. This can be explained through two main scenarios: simple retransmission attacks and assuming TLS compromise. When an attacker attempts to confuse the system by retransmitting a previously captured packet, *MQTree* calculates the hash value of every firmware file within the vehicle and stores it in the Merkle tree, thus generating a root hash. By comparing this root hash during the update process, the system prevents the misidentification and re-application of updates that have already been applied. This process effectively blocks attempts at duplicate updates, thereby protecting the system’s integrity. Even in cases where TLS is compromised, *MQTree’s* security mechanism continues to play a vital role. Even if encryption communication is threatened due to TLS damage, Merkle tree-based integrity verification ensures the safety of update packets. Even if the firmware’s hash and the root hash are altered, the root hash included in the update packet is ultimately compared with the root hash generated within the vehicle. Consequently, if damaged firmware is stored in SecureStorage, the system can roll back or delete it to eliminate potential security threats. This process is crucial for maintaining the system’s security, even in the event of TLS damage. In both scenarios, the logs generated during the verification process can be utilized by intrusion detection systems (IDSs), providing important information about the security status. However, mechanisms such as TLS and Merkle trees do not provide sufficient defense against DoS attacks on *MQTree*. Given that these attacks target to deplete system resources, additional security measures, such as firewalls and DDoS mitigation techniques, are required.

### 5.2. Performance Validation

In order to evaluate the efficiency and applicability of *MQTree* and explore ways to improve performance reflecting the latest technology trends, the performance validation was divided into two sections, as follows:

- Comparison with centralized network protocols: in order to provide a benchmark for *MQTree*, we selected MQTT with TLS and HTTPS and MQTT without TLS for comparison. This choice was based on existing research findings, such as those from various studies [6,25], indicating that MQTT with TLS outperforms HTTPS. The objective was to demonstrate that this study’s results obtained in the experimental environment align with the observed trend. The comparison between MQTT with TLS and HTTPS analyzed the differences in performance among widely used communication protocols to clarify the advantages and disadvantages of *MQTree* in relation to these standard protocols. Essentially, this comparison established that the results of this study conform to a general performance pattern.
- Comparison with blockchain protocol: In order to address contemporary technology trends, we evaluated the performance of MQTT systems integrated with the blockchain protocol. This exploration explores novel approaches that can improve the performance of the MQTT protocol. It is important to understand how the application of blockchain affects MQTT systems and its real-world implications. Consequently, a comparative analysis was conducted to evaluate MQTT-A and traditional MQTT (specifically MQTT with TLS) alongside blockchain sharding technologies while considering the impact of broker choice (e.g., HiveMQ and Mosquitto) on the performance of MQTT systems. This comprehensive comparison highlights the roles played by blockchain technology and broker choice in shaping the performance of MQTT systems.

#### 5.2.1. Comparison with Centralized Network Protocols

**Figure 7:**
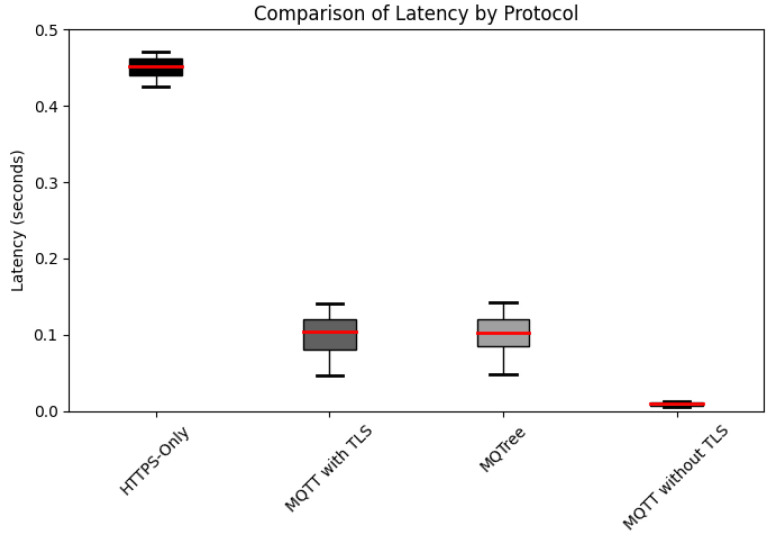
Comparison of the experimental results for latency. Note that the red line represents the average, and the squares represent the distribution of min and max values.

Figure 7 illustrates the performance measurements derived from 10 independent transactions with average latency calculations. The experimental setup incorporated Spring Boot version 2.7.7 for the server framework, with the server itself powered by an AMD Ryzen Threadripper PRO 5965WX CPU and 256 GB of RAM. On the client side, a system equipped with a 12th Gen Intel Core i7-1260P CPU and 16GB of RAM was used, running Python. The network configuration was established through a router supporting 100 Mbps bandwidth. For MQTT communication, Mosquitto version 2.0.11 served as the broker, and the Eclipse Paho MQTT client version 1.2.5 was utilized as the client library. The JSON payload adhered to a lightweight and efficient standard data format. Conveying the necessary information in OTA communication makes a notable contribution to saving network bandwidth and improving data transmission and processing speed. QoS was set to a default value of 1 in our experiments. The obtained results reveal an average latency of 0.467 s with HTTPS, 0.116 s with MQTT with TLS, and 0.0092 s with MQTT without TLS. Notably, MQTT with TLS demonstrated approximately four times greater speed than HTTPS.

Paris et al. [25] noted latency variations in the MQTT protocol when implementing QoS-1, with and without TLS. For example, at a QoS-1 level of two messages per minute, latency was 0.111898 microseconds without TLS, whereas with TLS, it increased to 39.87122 microseconds, an increase of approximately 356 times. Conversely, when transmitting 10,000 messages per minute, latency increased from 248.3012 microseconds to 36,132.43 microseconds with TLS, an increase of approximately 145 times. Similarly, Tang et al. [6] found that MQTT with TLS outperformed HTTPS by approximately 1.33 times, based on tests spanning QoS levels 0 to 2. In a single round-trip, MQTT without TLS and HTTP exhibited comparable durations, but MQTT with TLS required approximately 1.71 times longer. However, in a 10-round-trip test, MQTT with TLS was 1.33 times faster than HTTPS. Across various message payload lengths, MQTT demonstrated an average speed advantage of 1.25 times faster. This implies superior performance compared to an OTA system using the traditional Rest API proposed by Chandra et al. [15].

Given these comparisons, MQTT with TLS demonstrates a performance advantage over HTTPS, which makes it a robust alternative for satisfying security requirements without compromising performance. Although it may slightly trail behind in certain performance aspects compared to ciphers, such as AES, this consideration is pivotal from scalability and practicality perspectives. Notably, MQTT with TLS offers the advantage of easy integration without requiring significant modifications to the existing network architecture. The performance difference between MQTT and HTTPS can be attributed to MQTT’s lightweight and efficient message-handling protocol. MQTT requires less overhead for data transfer, and its performance advantage is particularly evident when using lightweight data formats. Collectively, these factors explain the reason why MQTT with TLS outperformed HTTPS in this experiment.

#### 5.2.2. Comparison with the Blockchain Protocol

Akshatha et al. [23] reported that the average latency of an MQTT system with blockchain sharding at the QoS-1 level ranges from approximately 0.04 ms to 0.22 ms. In contrast, MQTT with TLS communication exhibits an average latency of approximately 0.52 ms. This indicates that MQTT with TLS maintains a lower average latency of approximately 0.3 ms to 0.5 ms compared with the proposed method. In addition, in the study by Akshatha et al. [23], using HiveMQ at QoS-0, MQTT-A with TLS incurred a delay of 192 ms, whereas the proposed system demonstrated a reduced delay of 126 ms. This indicates that the sharding-based system outperforms MQTT-A with TLS by approximately 1.35 times. In their study using HiveMQCloud, Buccafurri et al. [26] observed that the transmission rate of MQTT with TLS and the proposed MQTT-A with TLS are closely aligned when considering the number of useful information bits received by the subscriber in a unit of time. Furthermore, it outperformed traditional MQTT with TLS by more than 3–5 times at QoS-0 and QoS-2.

**Figure 8:**
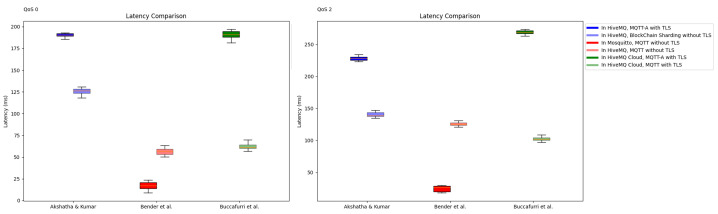
Comparison of experimental results based on broker choice. The studies compared in the experiment are as follows: Akshatha et al. [23], Bender et al. [27], and Buccafurri et al. [26].

However, a crucial distinction lies in the fact that the resource usage and performance of the MQTT protocol significantly hinge on the broker selected. In this study, we employed the Mosquitto broker, whereas other studies opted for HiveMQ. According to Bender et al. [27], the library can be efficiently implemented on resource-constrained IoT devices with fewer CPU cycles and less system memory usage, even when handling a substantial influx of incoming messages. This underscores that the resource usage and performance of the MQTT are notably dependent on the choice of broker, as shown in Figure 8. Resource usage tests revealed that the Mosquitto broker consistently exhibited the lowest CPU and memory requirements. In alignment with the experimental environment in this study, Mosquitto demonstrated the lowest average latency compared with other libraries, with the average latency at QoS-1 approximately five times higher than that of HiveMQ. These results highlight the substantial influence of broker choice on the overall performance of an MQTT system. In conclusion, it is essential to emphasize that even when using a centralized network instead of a blockchain, performance improvement is achievable through the optimization of MQTT’s broker selection.

An MQTT system that integrates blockchain technology using the optimization strategy proposed by Gao et al. may outperform the *MQTree* proposed in this study, depending on the broker choice. However, in practical scenarios where organizations seek to enhance security while seamlessly integrating with existing infrastructure, *MQTree* proves to be a viable solution. It fulfills these requirements effectively, delivering commendable performance and security results while maintaining compatibility with existing systems. In addition, the lightweight nature of the MQTT protocol optimizes the use of network resources, which is particularly valuable in resource-constrained scenarios, such as IoT environments. Therefore, *MQTree* emerges as a realistic and practical solution for simultaneously enhancing security and performance while enabling flexible integration with existing systems.

## 6. Discussion

*MQTree* is useful and effective for automotive software update; however, there are still avenues for future improvement. This section presents the limitations of the current *MQTree* system and their workarounds and discusses enhancements for future work.

### Discussion of Server-Side Security Assumptions

In this paper, we have focused our attention on the security of the client-side mechanism, particularly the integrity of firmware updates and the prevention of client-side attacks. We have assumed a secure server environment as the basis for our analysis, which is a common approach in the literature when delineating the scope of security assessments. We acknowledge, however, that in the real-world scenario, server-side security is of critical importance. A compromise of the server-side root hash could indeed lead to serious vulnerabilities, including persistent verification failures and impediments to the update process. While such considerations are beyond the scope of this current work, they represent a significant area for future research.

The potential for a discrepancy between server-side and client-side Merkle root hashes due to a server compromise raises important questions about the overall security posture of update systems. It is crucial for future systems to incorporate robust server-side defense mechanisms to mitigate such risks. This might include but is not limited to the use of certificate pinning, cross-verification protocols, and anomaly detection systems to safeguard the integrity of the server-side root hash. We suggest that subsequent work could build upon our findings by exploring these server-side security measures in detail. By doing so, the research community can develop a more holistic understanding of the security of update systems that encompasses both client and server perspectives.

## 7. Conclusions

This study has successfully demonstrated the application of Merkle trees to ensure firmware integrity for automotive over-the-air (OTA) updates within a centralized architecture. By employing MQTT with the TLS protocol alongside the traditional HTTPS approach, we have proposed a more efficient system that contributes to enhancing the reliability and efficiency of the firmware update process. It is important to note that our analysis assumes the security of the server environment, focusing on client-side verification to prevent attacks on the firmware update process. We recognize the critical nature of server-side security; however, an in-depth analysis of server-side vulnerabilities, including the compromise of the server-side root hash, is beyond the scope of this paper. This paper lays the groundwork for future research to explore these server-side security challenges.

As we look to the future, we aim to build upon the findings of this study by addressing the problem of a single point of failure through server redundancy and DDoS countermeasures. Further scenarios to determine the stationary status of vehicles will be investigated to enhance update safety. We also plan to develop advanced algorithms for Merkle tree performance optimization, which is expected to improve the efficiency of the entire system significantly. Through continuous improvements and the addition of robust server-side security measures in future work, the automotive OTA update system will evolve to provide an even more secure and efficient vehicle communication environment. This commitment to advancing OTA update mechanisms ensures that the system will remain resilient against a wide range of cyber threats, safeguarding the integrity of automotive firmware updates.

## Figures and Tables

**Table 1:** Major Attacks for OTA updates.

Attack	Description
Spoofing	An attack that involves an attacker impersonating another user or system with the intent to illegally obtain or transmit data.
MITM	An attack that intercepts the communication process and either tamper with or eavesdrops on messages.
DDoS	An attack that depletes a system’s resources and disrupts services.
Duplicate Update	An attack that disrupts the normal operation of a system by allowing an attacker to resend an update message that has already been sent.

**Table 2:** Summary of related work in automotive OTA update systems.

Research	Key Findings/Methodologies	Area	Focus
Gemirter et al. [11]	Found MQTT to have significantly shorter message transfer times and lower CPU utilization than HTTP, showcasing MQTT’s efficiency in message delivery and resource utilization.	Reliability and Efficiency of Communications	Utilization of MQTT for efficient data transfer.
Luzuriaga et al. [12]	Demonstrated MQTT’s capability to maintain message delivery order in unstable environments, such as vehicular networks, which is crucial for sequential packet reception in OTA updates.		
Thantharate et al. [13]	Showed that MQTT has a shorter message delivery time than constrained application protocol (CoAP), even under limited RF conditions, indicating the high transmission efficiency of MQTT under various network conditions.		
Al-Fuqaha et al. [14]	Found that MQTT experienced lower delays than CoAP under low packet loss conditions, highlighting the stability and reliability of MQTT for fast data transfer.		
Sahlmann et al. [9]	Proposed slicing the firmware file for efficient transfers and maintaining integrity by providing the firmware file’s location as a URL in JSON format, enhancing data security through secure downloading over HTTPS.		Methods to maintain integrity and prevent data loss in OTA updates.
Chandra et al. [15]	Focused on securely downloading URLs over the HTTPS protocol to enhance data security in IoT environments.		
Stoev et al. [16]	Used a 256 bit symmetric encryption algorithm (AES256 ECB mode) to enhance the security of MQTT protocol communication, despite vulnerabilities to replay attacks and the generalization problems of the security mechanism.		
Tsaur et al. [17]	Proposed a highly secure IoT firmware update mechanism using blockchain to enhance security and reliability.	Security Enhancements	Decentralized approaches using blockchain for firmware integrity and authenticity.
Falco et al. [18]	Employed distributed hash tables and blockchain to ensure data and software integrity in connected vehicles.		
Buccafurri et al. [19]	Focused on securing the MQTT protocol for IoT devices using blockchain-based OTP authentication to enhance security.		
Aknin et al. [20]	Proposed an architecture using Ethereum blockchain and smart contracts to secure MQTT architecture and enhance transaction security.		
Abdelrazig et al. [21]	Developed a blockchain-based identity and authentication scheme for the MQTT protocol to address basic security vulnerabilities.		
Gao et al. [22]	Introduced a blockchain-based MQTT protocol optimization algorithm for improved security and efficiency.		
Akshatha et al. [23]	Explored user-controlled data access with improved security and efficiency through MQTT and blockchain sharding.		
Da Silva et al. [24]	Proposed a secure OTA approach using TLS to enhance the operation and security of emergency detection units in smart cities.		

**Table 3:** Related studies for comparison.

OTA Type	Description	Study
HTTPS Only	Verify the security of OTAs using the traditional security protocol HTTPS (or TLS). In particular, we will discuss how HTTPS can respond to each security threat and its limitations.	Chandra et al. [15]
MQTT with TLS	Analyze the benefits and limitations of applying TLS security features to the MQTT protocol.	Stoev et al. [16]
MQTT without TLS	Explore various solutions for securing the MQTT protocol using alternative methods of secure communication instead of TLS and analyze their benefits and limitations.	Sahlmann et al. [9] Da Silva et al. [24]

**Table 4:** Comparative study based on security threat.

Study	Dos/DDoS	MITM	Spoofing	Duplicate Update
*MQTree*	X	O	O	O
Chandra et al. [15]	X	O	O	X
Stoev et al. [16]	X	O	X	X
Sahlmann et al. [9]	X	X	X	X
Da Silva et al. [24]	X	O	O	X

## Data Availability

The data (source code) used in this study can be found here: https://github.com/SYunje/MQTree, accesed on 19 February 2024.

## Abbreviations

The following abbreviations are used in this manuscript:

SDV	Software-Defined Vehicle
OTA	Over the air
FOTA	Firmware over the air
MQTT	Message queuing telemetry transport
AMQP	Advanced message queuing protocol
QoS	Quality of service
V2X	Vehicle to everything
IoT	Internet of things
ECU	Electronic control unit
MITM	Man in-the-middle attack
DDoS	Distributed denial of service
DoS	Denial of service
HSM	Hardware security module
MFA	Multi-factor authentication
PoLP	Principle of least privilege
SIEM	Security information and event management
CoAP	Constrained application protocol
CLI	Command-line interface
MUP	MYNO update protocol
IDS	Intrusion detection system
